# Burden of nursing care: a concept analysis

**DOI:** 10.15649/cuidarte.3848

**Published:** 2024-09-19

**Authors:** Lina María Vargas-Escobar, Kevin Julián Aya-Roa, Judith Liliana Ortiz-Mayorga, Marcia Andrea Quiñonez-Mora, Laura Marcela Hernández-Bohórquez, Genny Paola Fuentes-Bermúdez, Alexander Casallas-Vega

**Affiliations:** 1 Full Professor. Faculty of Nursing, Universidad El Bosque. Bogotá, Colombia. E-mail: lmvargase@unbosque.edu.co Universidad El Bosque Faculty of Nursing Universidad El Bosque Bogotá Colombia lmvargase@unbosque.edu.co; 2 PhD student in Nursing Sciences, Celaya- Salvatierra Campus, Universidad de Guanajuato, Celaya, Mexico. E-mail: kj.ayaroa@ugto.mx Universidad de Guanajuato Celaya- Salvatierra Campus Universidad de Guanajuato Celaya Mexico kj.ayaroa@ugto.mx; 3 Director of Nursing, Los Cobos Medical Center. Bogotá, Colombia. E-mail: jlortizm@loscobosmc.com Los Cobos Medical Center Bogotá Colombia jlortizm@loscobosmc.com; 4 Administrative Head of Intensive Care Units. Los Cobos Medical Center. Bogotá, Colombia. Email: maquinonezm@unal.edu.co Los Cobos Medical Center Bogotá Colombia maquinonezm@unal.edu.co; 5 Administrative Head of Hospitalization and Support Services, Los Cobos Medical Center. Bogotá, Colombia. E-mail: lmhernandezb@loscobosmc.com Los Cobos Medical Center Bogotá Colombia lmhernandezb@loscobosmc.com; 6 Profesora medio tiempo, Facultad de Enfermería, Universidad El Bosque. Bogotá, Colombia. E-mail: gfuentesb@unbosque.edu.co Universidad El Bosque Facultad de Enfermería Universidad El Bosque Bogotá Colombia gfuentesb@unbosque.edu.co; 7 Assistant Professor. School of Nursing, Universidad El Bosque, Bogotá, Colombia. E-mail: acasallasv@unbosque.edu.co Universidad El Bosque School of Nursing Universidad El Bosque Bogotá Colombia acasallasv@unbosque.edu.co

**Keywords:** Nursing, Nursing Research, Concept Formation, Nursing Theory, Patient Care Planning, Enfermería, Investigación en Enfermería, Formación de Concepto, Teoría de Enfermería, Planificación de Atención al Paciente, Pesquisa de Enfermagem, Formação de Conceito, Teoria de Enfermagem, Planejamento de Assistência ao Paciente.

## Abstract

**Introduction:**

The burden of nursing care has not been defined as a theoretical or operational concept. However, several studies have demonstrated its impact on the quality of care, the increase in adverse events, and the health of nursing staff.

**Objective:**

To analyze the attributes, factors, antecedents, and consequences associated with the concept of 'burden of nursing care' in order to clarify its meaning.

**Materials and Methods:**

A concept analysis was conducted using Walker and Avant's methodological proposal, which examines a concept's defining characteristics and attributes.

**Results:**

The burden of nursing care is the relation between the needs of patients and the time available for direct care, management, and education activities. Both intrinsic and extrinsic patient factors can influence the patient's level of dependency and needs, increasing nursing interventions and hours of care, thereby intensifying the burden of care.

**Discussion:**

The burden of nursing care involves patient centered care with adequate resources. The burden of nursing care demands planning and leadership from nurses. A lack of competencies and experience, combined with institutional inflexibility, increases the burden of nursing care, leading to dissatisfaction and adverse events.

**Conclusions:**

The burden of nursing care is a concept that is epistemically grounded in the interactive-integrative view because it focuses on meeting patients' needs through nursing interventions for direct care, management, and education.

## Resumen

**Introducción:**

La carga del cuidado de enfermería no se ha definido como concepto teórico ni operacional; sinembargo, diversos estudios han demostrado su impacto en la calidad de la atención de enfermería, el incremento de eventos adversos y la salud del personal de enfermería.

**Objetivo:**

Analizar atributos, factores, antecedentes y consecuencias vinculadas al concepto de Carga del Cuidado de Enfermería con el fin de esclarecer su significado.

**Materiales y Métodos:**

Análisis de concepto a partir de la propuesta metodológica de Walker y Avant, a través de la cual se examinan las características que define un concepto con sus atributos.

**Resultados:**

La carga del cuidado de enfermería es la relación entre las necesidades de los pacientes y el tiempo disponible para actividades directas, de gestión y educación. Tanto los factores intrínsecos como los extrínsecos del paciente pueden influir en su nivel de dependencia y necesidades, incrementando las intervenciones de enfermería y las horas de cuidado, impactando así la carga del cuidado.

**Discusión:**

La carga de cuidado de enfermería implica atención centrada en el paciente con recursos adecuados. Requiere planificación y liderazgo por parte del profesional de enfermería. La falta de competencias y experiencia, junto con la inflexibilidad institucional, aumenta la carga y conduce a insatisfacción y eventos adversos.

**Conclusiones:**

La carga del cuidado de enfermería es un concepto con fundamentación epistémica en la visión interactiva-integrativa, dado que se centra en la satisfacción de las necesidades de los pacientes mediante las intervenciones de enfermería de cuidado directo, de gestión y educación.

## Resumo.

**Introdução:**

A sobrecarga dos Cuidados de Enfermagem não foi definida como um conceito teórico ou operacional, no entanto, existem vários estudos que demonstram o seu impacto na qualidade dos cuidados de enfermagem, no aumento de eventos adversos e na saúde dos trabalhadores de enfermagem.

**Objetivo:**

Analisar atributos, fatores, antecedentes e consequências vinculados ao conceito de Sobrecarga de Cuidados de Enfermagem para esclarecer seu significado.

**Materiais e Métodos:**

Análise de conceito baseada na proposta metodológica de Walker e Avant, por meio da qual são examinadas as características que definem um conceito com seus atributos.

**Resultados:**

A sobrecarga do cuidado de enfermagem é a relação entre as necessidades dos pacientes e o tempo disponível para atividades diretas, gerenciais e educativas. Tanto os fatores intrínsecos quanto os extrínsecos do paciente podem influenciar seu nível de dependência e necessidades, aumentando as intervenções de enfermagem e as horas de cuidado, impactando assim na carga de cuidado.

**Discussão:**

A sobrecarga de cuidados de enfermagem implica cuidados centrados no paciente e com recursos adequados. Requer planejamento e liderança por parte do profissional de enfermagem. A falta de competências e experiência, aliada à inflexibilidade institucional, aumenta a carga e leva à insatisfação e a eventos adversos.

**Conclusões:**

A carga dos cuidados de enfermagem é um conceito com fundamentos epistêmicos na visão interativo-integrativa, dado que se concentra na satisfação das necessidades dos pacientes por meio de intervenções diretas de cuidado, gestão e educação de enfermagem.

## Introduction

The burden of nursing care has not been defined as a theoretical or operational concept but there are other concepts, such as caregiver burden, that can contribute to its construction. Caregiver burden is defined as the level of multifaceted strain individuals perceive from caring for a family member and/or loved one over time^1^. This concept was intended for caregivers of individuals with chronic illnesses. Additionally, the concept of formal caregiving burden in nursing homes has been defined as the demands of caring for dependent older adults with a specific level of competence and responsibility within a context of perceived stress^2^.

A third concept is workload, defined as the set of demands placed on people during their working day^3^. This concept makes the greatest contribution to the construction of the concept of 'burden of nursing care.' Nursing workload affects the health of nurses and the quality of care. Studies like the one by Nantsupawat et al. in Thailand found that a higher workload was associated with increased stress, fatigue, and job dissatisfaction among nurses^4^. On the other hand, Bordignon and Monteir's study in Brazil revealed that an excessive and unbalanced workload was associated with a higher intention to leave the profession and lower job satisfaction^5^.

The increased burden of care has been identified as a critical factor in increased adverse events, infections, hospital readmissions, and negative clinical outcomes for patients^6^^, ^^7^. The high number of patients assigned to each nurse is associated not only with increased patient mortality, but also with hospital readmissions and longer hospital stays^8^^, ^^9^.

From the perspective of the burden of nursing care, the subjects of care are regarded as holistic beings with biological, psychological, sociocultural, and spiritual components. This concept demonstrates that one of the main functions of nursing care is to meet the needs that patients may have while they are hospitalized in a healthcare facility. According to Virginia Henderson's theory, for people to achieve and/or regain their health condition, it is necessary that all of their needs are continuously met^10^.

By satisfying needs through nursing care provided in health institutions, individuals facing illness can recover their health, well-being, and harmony^11^. This distinguishes the burden of nursing care from the nursing workload. The burden of nursing care considers all patient needs, both basic needs and hospital management and educational needs, taking into account direct care, education, and management activities. The nursing workload focuses on the number of hours or minutes of direct care and is defined as the time required to provide direct and indirect care to a person in a vulnerable situation^12^.

This concept analysis is relevant because, in the literature review conducted by the researchers, no concept was identified that explains how the burden of care defines the complexity and quantity of interventions, as well as the direct, managerial, and educational activities that nurses must perform based on the specific needs of subjects of care in hospital inpatient settings. A concept that explains the burden of care from a perspective focused on the nursing care tasks rather than the working conditions under which these interventions occur.

Therefore, the concept of 'burden of nursing care' is necessary to more accurately assess the demands on nursing staff in hospital services, from a holistic view that considers all of the patients’ needs and the amount and complexity of interventions required for each user during the service, and not from a view of compliance with only direct care or the particular deterministic paradigm.

Nursing care burden is the set of responsibilities and tasks that a nurse must perform to ensure comprehensive and quality care for hospitalized individuals. The bill, which repeals Law 911 of 2004, emphasizes the importance of managing these responsibilities efficiently and safely, ensuring that nurses have the necessary resources, such as sufficient personnel, adequate time, and optimal working conditions, to deliver effective care. This not only enhances the quality of care but also safeguards the health and well-being of nurses, allowing them to perform their duties with the diligence and professionalism that society demands.

This concept offers a more specific and quantifiable perspective on care demands in relation to available resources. This concept analysis enables a more precise identification of areas for improvement in resource allocation and staffing planning. Theoretically, the concept of 'burden of nursing care' offers a disciplinary conceptual framework for developing measurement instruments to assess the level of nursing care burden in inpatient settings. In addition, it makes visible nursing activities that are often unrecorded and, therefore, invisible, such as educational and managerial activities^13^. Practically, analyzing this concept can inform policies and practices that enhance working conditions, reduce stress and burnout, and ultimately lead to better health outcomes for patients. For this reason, the objective of this study is to analyze the attributes, factors, antecedents, and consequences associated with the concept of burden of nursing care to clarify its meaning.

## Materials and Methods

For analyzing the concept of burden of care, we used Walker and Avant's proposal^14^, which suggests that concept analysis is a method for developing theories and offers the opportunity to explain and describe phenomena of interest for practice. It is a strategy for examining the characteristics and attributes that define the concept, and that makes it possible to decide which phenomenon exemplifies the concept better and which does not through the identification of a model case. This method consists of eight steps, but for this study, we used seven: 1) selection of the concept, 2) description of the objectives or purposes of the analysis, 3) identification of the uses of the concept, 4) identification of the characteristics or defining attributes, 5) identification of a model case, 6) identification of antecedents and consequences, and 7) definition of empirical referents.

The first step was to select the concept 'burden of nursing care,' and the second was to determine the purpose of the analysis. The third step is to identify and clarify the use of the concept. For this purpose, an intentional search of the literature was conducted, which seeks to find definitions, research, official documents and others that allow the identification of similar concepts that could help clarify the selected concept, starting from the most general to the most concrete and disciplinary found. This approach allows for the extraction of conceptual elements and the differentiation of the selected concept from those identified in the intentional review. In the fourth step, the characteristics or defining attributes of the burden of nursing care are described. In order to clarify the characteristics or defining attributes of the concept, an exhaustive analysis of the studies found in the intensive literature review was conducted. In addition, an expert meeting was held with the participation of the researchers of this study and professional nurses from various higher education and healthcare institutions in Bogotá. During this meeting, findings from the literature were presented, professional experiences were shared, and key attributes of the concept 'burden of nursing care' were identified.

In the fifth step, we describe a model case that illustrates the burden of nursing care in an internal medicine inpatient service. In the sixth step, we describe the antecedents -actions or conditions necessary for the concept to occur; the consequences -results of the event’s occurrence; and the factors that might influence the burden of nursing care^14^. Some elements of the possible empirical referents are described in step seven.

The intentional search was carried out between January and June 2023 in different databases, such as PubMed, Scopus, Embase, EBSCO, ScienceDirect and Springer, using health science descriptors (DeCS) and Boolean operators in English and Spanish, such as workload (carga de trabajo) AND nursing (enfermería) AND hospitalization (hospitalización) OR inpatient care units (unidades de internación). The data from the studies supporting the following concept analysis are available in Mendeley Data for free access and consultation^15^.

## Results

The following outlines the development of the seven steps of concept analysis according to the selected method:

### 1. Concept selection

The concept to be analyzed is the 'burden of nursing care.'

### 2. Selection of the purpose of the concept analysis

The importance of selecting the concept of 'burden of nursing care' lies in the possibility of assessing the responsibilities nurses face in their daily practice, including direct care, education, and administrative activities, as well as the time required to perform these tasks with quality. Defining the concept of 'burden of nursing care' could provide evidence to help understand how nursing professionals invest time to perform their professional duties and, in this way, advance towards the development of future research to establish indicators and mathematical formulas to calculate the number of nursing professionals needed to provide care for a given number of patients in different care units.

### 3. Clarification and identification of the uses of the concept

Currently, the concept of nursing care burden is not clearly defined, as the focus has primarily been on the burden of care experienced by informal, primary, or family caregivers^16^. This concept has been defined through three key attributes: self-perception, multifaceted strain, and the passage of time. Caregiver burden is determined by the caregiver's positive or negative perception of their caregiving responsibilities. In addition, caregiving can create strain at a multidimensional level, potentially affecting the health of caregivers. Finally, defining caregiver burden requires understanding that the phenomenon is not static and can change significantly over time^1^.

Another relevant concept is the formal caregiving burden in nursing homes, which implies a level of competence and responsibility to meet the demands of caring for dependent individuals, within a context of perceived stress. The presence of this concept depends on organizational and environmental conditions, and its consequences are reflected in changes in the physical and mental health of caregivers^2^.

Conversely, workload has been defined as “the set of mental and physical demands placed on professionals during their workday” ^17^.Workload can be viewed as the interaction between the demands of job performance and an individual's own capabilities to fulfill their duties. This can be understood quantitatively by the number of tasks to be completed within a given time and qualitatively by the complexity of the activities and the resources needed to perform them^18^. Therefore, workload can be understood as the physical and mental effort required to complete specific work activities within a given time.

Schmoeller et al. ^19^ define nursing workload as the product of the average daily number of patients seen, adjusted by the degree of dependence and type of care and the average time of assistance for each patient. A concept analysis conducted by Alghamdi^20^ in 2016 defined nursing workload as the amount of physical exertion related to time spent caring per patient, level of nursing competency, direct patient care, and complexity of care. This exertion is directed toward direct and indirect activities, as well as activities unrelated to patient care.

As a professional discipline focused on human care, life, and health, nursing involves a continuous effort to provide comprehensive care to patients. This responsibility is reflected in labor terms as the burden of nursing care.

An increased nursing workload leads to a reduction in patient survival rates, directly impacting the overall care of patients with higher care needs. Nurse understaffing is associated with the omission of essential care and adverse patient outcomes. Therefore, using the nurse-to-patient ratio as an indicator for determining nursing care burden is only one factor in this complex and abstract phenomenon^21^^, ^^22^.

Increased nursing care hours and activities for a specific patient are associated with a higher incidence of adverse events^23^. Nursing care for individuals with greater needs is a risk factor for adverse events such as pressure ulcers and medication errors, as addressing the needs of one patient with greater care requirements may lead to neglecting the care of other patients^16^^, ^^23^.

The concept of 'burden of nursing care' involves recognizing the amount and complexity of care required for each patient during their hospital stay. This includes factors such as the illness's severity, comorbidities, the patient's level of dependence, and the intensity of treatments. To address these requirements, nursing activities and interventions must be implemented to meet the patient's needs, which in turn affect the workload that nurses have to face.

The caregiving burden for caregivers of chronically ill individuals is defined as all the demands, functions, and activities carried out by the caregiver^16^. In this sense, the concept of 'burden of nursing care' could serve as a conceptual basis for determining the nurse-to-patient ratio in a specific care unit by identifying care needs, patient dependency levels, and nursing activities.

From the concept of 'burden of nursing care,' the subject of care is an individual hospitalized in a healthcare facility due to a health condition and receiving care from nurses and a multidisciplinary team. The subjects of care also include the family members or family caregivers who assist and participate in the patient's care. In this way, the subjects of care are viewed holistically as an integrated whole that cannot be reduced to purely physical aspects^24^. The concept of 'burden of nursing care' is based on the reciprocal-interaction view proposed by Newman^25^^, ^^26^ or the interactive-integrative view proposed by Fawcett^27^, since the burden of nursing care in services such as hospitals leads nurses to perform direct care, management, and educational functions^28^.

The reciprocal-interaction or interactive-integrative paradigm enables a holistic view of the subject of care, guiding nurses to assume a role and focus their functions on fully and comprehensively meeting the subject of care's needs. This means that nurses are not solely focused on the sick physical body but also acknowledge other dimensions such as mental, familial, and spiritual well-being^29^. In addition, the administrative and care management components that ensure the provision of direct care to satisfy the subjects of care's needs in the various dimensions are considered.

Nursing workload refers to the total volume of work a nurse must perform, encompassing direct patient care tasks, administrative responsibilities, resource management, coordination with other healthcare professionals, and training and professional development activities. In contrast, the burden of nursing care, defined as the relationship between the care needs of patients and the nurse's available time to provide direct care, management, and educational activities, focuses specifically on direct patient interaction. The burden of nursing care is influenced by factors such as the complexity of the patient's condition, the number of interventions required, and the time needed to provide quality care. While workload encompasses all the responsibilities inherent in the nurse's role, the burden of nursing care focuses on the time and effort dedicated directly to meeting patients' needs, emphasizing the critical relationship between available resources and the demand for care.

On the other hand, from an interactive-integrative perspective, context plays a fundamental role in the way care is provided. The subject of care and the context interact all the time, making it possible to generate changes that contribute to achieving different levels of health. In this sense, educational aspects and interventions to support and assist the subject of care are essential for addressing their care needs and should be integrated into the daily activities of nursing care^24^^, ^^25^.

### 4. Defining attributes of the concept

The following attributes of the burden of nursing care were identified:

- Care needs (physical, psychological, social, and spiritual).
- Time and resources available to nurses.
- Direct care, management, and education activities.

### Care needs (physical, psychological, social, and spiritual)

Physical needs are a crucial aspect of the concept of 'burden of nursing care,' encompassing requirements such as patient mobility, hygiene, and nutrition. These physical demands, if not adequately addressed, can significantly impact the burden of nursing care and the nurse's ability to provide care^30^.

It has been found that the greater the patients' physical needs, based on the severity oftheir condition, the greater the nurses' burden of care. Similarly, emotional challenges, stress, or anxiety can increase nurses' burden, as they require an additional approach to providing emotional support and care^31^.

Psychological needs are another essential attribute. A patient's mental health, emotional well-being, and psychological stability have a direct impact on the burden of nursing care. Taken together, these physical, psychological, social, and spiritual needs represent interrelated attributes of the concept 'burden of nursing care,' each of which can influence and affect the overall care experience for both patients and nurses.

### Time and resources available to nurses

The nurse's available time is a critical attribute within the 'burden of nursing care' concept. This essential factor refers to the tangible and available time that nursing staff can dedicate to addressing patients' needs.

The time required to provide quality care should reflect the specific needs of patients and be accurately determined for the multiple responsibilities of the nursing team. Time availability directly affects the ability of nursing staff to provide direct care, management, and education to users and family members. Time constraints may make it difficult to perform these functions properly. Various factors influence this attribute, including patient assignment, the complexity of patient conditions, available resources, and healthcare policies^32^.

When these factors limit the time available, the burden of nursing care increases, affecting the quality of care provided and the satisfaction of both nurses and patients. A systematic review found that nurses face a significant burden of care, primarily due to the amount of time spent caring for each patient according to their needs, which often exceeds the time available for direct care. This situation disrupts the balance of nursing work and increases the workload^33^.

A study revealed that the significant increase in time spent on direct care, education, and management activities is often associated with a lack of nursing staff. This situation arises because the number of nursing activities required per patient exceeds the available physical capacity^34^.

A longitudinal study conducted in the Netherlands^35^ indicates that the burden of nursing café models the job resource support of colleagues and job demands. Time spent on direct patient care and time spent on registration had the biggest significant effects on perceived workload in that study. Resource management in the service unit can help reduce the perception of burden by facilitating the nursing team to work together smoothly and enhancing team spirit.

Professional and institutional resources are critical components of the burden of nursing care. The balance between these resources and nursing activities is essential to meeting patient needs. When nursing care activities exceed available resources or when those resources are insufficient, it is difficult to meet the actual needs of patients and their families. This increases the burden of nursing care and work pressure on nurses^36^.

### Direct nursing care, management, and education activities

Direct nursing care, management, and educational activities directly impact the burden of nursing care. In direct care, addressing patients' physical and emotional needs can either increase or decrease the burden of care, depending on the available time and professional competencies of the nursing staff. Management activities, such as resource organization and service coordination, can also influence the burden of care; effective management can optimize available processes and resources, thereby reducing the burden on nursing staff. On the other hand, patient and family education can alleviate long-term care burden by empowering patients and their families to take better care of themselves, even though it may initially demand more time and effort from the nursing staff. Collectively, the quality and balance among these activities significantly influence the overall burden of care experienced by nurses^33^^, ^^34^.

Educational activities aimed at patients and their family members could impact the burden of nursing care. An increase in educational activities was observed at the beginning and end of hospitalization, significantly raising the burden during these two periods due to the time spent on them at the start of the hospital stay and at discharge^37^.

Changing dressings is one of direct nursing activity that increases the time spent and, consequently, the burden of nursing care. Patients who require multiple dressing changes during a shift contribute significantly to this burden^38^.

The number and complexity of nursing activities involved in providing direct care to certain types of patients, such as those with chronic kidney failure, significantly increase the workload^39^. This is because they require a series of specific actions, including preparation before, care during, and after the procedure. Nursing activities represent a significant dimension of workload, as some, like direct care or educational tasks, demand more personal resources and time than others. This differs from the general time available to nurses during their shifts to perform these functions.

### 5. Identification of a model case

The following is a true account of a nurse providing care in the internal medicine inpatient service:

One day, Oscar, a nurse working in a tertiary care clinic on an internal medicine service, is assigned 12 patients (in an inpatient service with a capacity for 30 patients). During the shift handover, Oscar notes that the care needs and demands of his 12 patients are greater than those of the other patients in the entire inpatient service. The patients under his care are mostly older adults with chronic underlying conditions, and three of them had been readmitted due to misuse of insulin at home and were scheduled to be discharged later that day. Oscar was responsible for designing these patients' hospital discharge plans, including insulin management education. On the other hand, two patients were at imminent risk of sudden death and had signed informed dissent, while the other three patients had various diseases, with high degrees of dependency according to the Barthel Index and processes of cognitive deterioration.

To manage these 12 patients, Oscar needed to distribute three nursing assistants among them to ensure that the number of activities performed during the shift was equal across all patients.

Oscar identified a higher burden of nursing care among his patients for that shift; however, according to the clinic policy, patient assignments are made based on bed order, ranging from beds 300 to 315 and 316 to 330. After the shift handover and nursing assessment, Oscar established priority activities for each patient. Based on his experience, he knew that no matter how efficiently he worked, neither he nor his night shift partner could meet all the necessary direct care, management, and educational interventions to address his patients' needs.

Oscar then requested an additional nurse for the service from the Nurse Care Coordination, along with a new patient assignment, due to the high nursing care demands of the patients during that shift. He made this request to efficiently address the direct care needs related to management, education, and the spiritual needs of both the family and patients at risk of sudden death, as well as other identified direct care needs.

Since Oscar justified the connection between patient care needs and the nurse's physical, mental, spiritual, and time capacities to provide direct care, management, and educational interventions, an additional nurse was assigned to cover patient needs, along with the support of a nursing assistant for that shift, ensuring an equitable distribution of the care burden.

### 6. Antecedents and consequences

**Figure 1:**
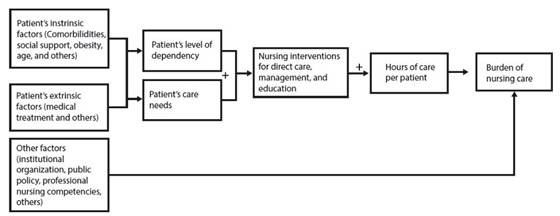
Factors influencing the burden of nursing care

As illustrated in Figure 1, there are three types of factors that can indirectly influence the burden of nursing care: intrinsic (personal) and extrinsic factors. Intrinsic factors, such as age, comorbidities, risk of sudden death, and obesity, increase the patient's level of dependency and needs, thereby influencing the burden of nursing care^40^^, ^^41^. On the other hand, extrinsic factors are outside the patient's control, such as medical treatment and the reason for hospitalization, which also impact the burden of nursing care^39^^, ^^41^.

Additionally, factors such as the nurses’ competencies, institutional factors, and public policies can directly influence the burden of nursing. In research conducted in Italy, multivariate regression models revealed that patient condition, nursing staff resources, patient transfers, documentation, patients in isolation, unscheduled activities, and patients from different specialties were significant predictors of nursing burden^42^. These factors increase patient dependency and care needs, thereby increasing both the number and complexity of nursing interventions, whether in direct care, management, or education. Consequently, this leads to a rise in the hours spent on care, thereby increasing the burden of nursing care.

Public policy influences the burden of nursing care by setting rules and regulations that determine staffing levels, care quality standards, resource allocation, and working conditions. For example, policies that mandate higher nurse staffing levels or limit the number of patients per nurse can reduce the burden of care, whereas policies that fail to address adequately these issues may increase it.

Comprehensive care is a nursing responsibility and includes taking on the burden of care. To do so, nurses must meet patients' needs and fulfill all direct care, management, and educational functions^43^. Direct nursing care activities, such as dressing changes, along with educational and management tasks like patient admissions and discharges, increase the hours spent on each patient, thereby intensifying the burden of nursing care^36^^, ^^37^^, ^^39^^, ^^40^.

For an optimal burden of nursing care, there must be a balance between the nurse’s physical, mental, spiritual, and time capacities, the care needs (physical, psychological, social, and spiritual), and the direct care activities, functions, and interventions, as well as the management and educational responsibilities.

**Figure 2:**
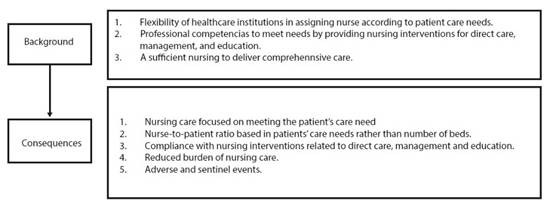
Background factors and consequences of nursing care burden

For the burden of nursing care to be optimal, there must be background factors that lead to specific consequences, as illustrated in Figure 2.

The nursing care model (assessment, diagnosis, planning, implementation, and evaluation) acknowledges that several factors, such as institutional, professional, multidisciplinary team, and patient factors, impact the type of care nurses provide during the planning phase. When nursing care does not adequately address the patient's physical, mental, spiritual, and social needs, holistic care is compromised. The omission or delay in addressing these needs, caused by various factors, leads to incomplete or insufficient care^44^^, ^^45^. The lack of holistic care resulting from missed nursing care and imbalance of nursing skills regarding patient needs and nursing interventions leads to negative outcomes in patient health, quality, and safety of care^39^.

To ensure an optimal burden of nursing care, institutions must be flexible in allocating nursing staff, considering the care needs of patients. In their research, Sir et al. ^46^ designed and tested various nurse-patient assignment models aimed at achieving a balanced workload distribution, considering patient acuity metrics. Mathematical models for staffing based on the characteristics and needs of the population can help improve nurses' working conditions and help them stay by reducing and balancing their workloads.

Institutions should maintain flexibility in assessing the daily burden of nursing care across different services, enabling the allocation of additional nurses to services with higher burdens at any given time. The burden of nursing care can fluctuate significantly between services throughout the day, so the nurse in charge of each service must promptly assess and adjust the care burden according to the patient's needs^47^.

### 7. Definition of empirical referents

Given its broad scope, the burden of nursing care requires the development of an instrument that can assess its various dimensions. These dimensions should encompass physical, psychological, social, and spiritual care needs, as well as the quantity and complexity of nursing interventions and activities, including direct care, management, and education. This instrument should be designed to align with the nurses' available time, adapting to the specific needs and interventions required in each case.

### Operational definition of the concept

Based on the above, the burden of nursing care can be defined as the relationship between the care needs of the subjects of care and the time available to the nurse to perform direct care, management, and educational activities and interventions.

## Discussion

The concept of 'burden of nursing care' suggests that nursing care is patient-centered, and the institutional, personal, and environmental resources are adequately provided to meet patients' needs without restriction. Therefore, nurses plan and lead direct care, management, and educational activities. For this to happen, flexibility in healthcare institutions, sufficient professional competencies, and human resources in nursing are required to meet all the needs of hospitalized patients.

The lack of professional competencies and clinical experience, coupled with healthcare institutions' inflexibility in providing supervision by experienced personnel, can result in adverse events due to increased administrative activities^43^. All these factors contribute to unmet health needs and an increased burden of nursing care.

By properly applying the burden of nursing care in hospital services, the nurse-to-patient ratio would be aligned with patients' care needs instead of being based solely on the number of beds. The assigned patients' care needs would then be addressed, along with the nursing activities related to direct care, management, and education. As a result, the burden of nursing care would be reduced. In many cases, nurses have to compensate for the shortage of personnel, both assistants and professionals, by delegating some specific functions and activities of nurses to nursing assistants in order to complete the shift in time and form^48^. Typically, the number of nursing activities and interventions exceeds the available nursing staff resources, and the time spent per patient is greater, often surpassing the burden of nursing care^34^.

## Conclusion

The concept of 'burden of nursing care' traditionally has a labor-related connotation. However, based on the attributes identified in the literature review, this phenomenon goes beyond a purely labor-related connotation. This concept includes dimensions that encompass patients' physical, psychological, social, and spiritual care needs in the hospital setting, as well as the time available to nurses to intervene through direct care, management, and education.

Factors and antecedents of nursing care burden influence the balance between patient needs and nurses' time available for direct care, management, and education. This balance ensures needs- centered care and results in less overburden for nurses.

In this context, it is crucial to distinguish the burden of nursing care from the workload. The burden of nursing care encompasses a broader, more holistic perspective, taking into account not only the workload but also the quality of care, and the overall well-being of both patients and nurses. This perspective requires a detailed and specific understanding of the concept, moving beyond generic recommendations to clearly identify the factors that distinguish it from mere workload.
